# Neopterin Levels in Bonobos Vary Seasonally and Reflect Symptomatic Respiratory Infections

**DOI:** 10.1007/s10393-023-01633-y

**Published:** 2023-05-15

**Authors:** Mélodie Kreyer, Verena Behringer, Caroline Deimel, Barbara Fruth

**Affiliations:** 1 Department for the Ecology of Animal Societies, Max-Planck Institute of Animal Behavior, Bücklestraße 5 a, 78467 Constance, Germany; 2 Centre for Research and Conservation, Royal Zoological Society of Antwerp, Antwerp, Belgium; 3 School of Biological and Environmental Sciences, Faculty of Science, Liverpool John Moores University, Liverpool, UK; 4 Endocrinology Laboratory, German Primate Center, Leibniz Institute for Primate Research, Göttingen, Germany; 5 Research Group Evolutionary Physiology, Max Planck Institute for Ornithology, Seewiesen, Germany

**Keywords:** *Pan paniscus*, Urinary neopterin, Health monitoring, Non-invasive, Respiratory infections, Ecoimmunology, Seasonal infectious diseases

## Abstract

As environmental changes exacerbate the threat coming from infectious diseases in wild mammal species, monitoring their health and gaining a better understanding of the immune functioning at the species level have become critically important. Neopterin is a biomarker of cell-mediated immune responses to intracellular infections. We investigated the variation of urinary neopterin (uNeo) levels of wild, habituated bonobos (*Pan paniscus*) in relation to individual and environmental factors. We used 309 urine samples collected between 2010 and 2018 at the LuiKotale field site, DRC. Based on current knowledge on zoo-housed conspecifics and closely related species, we predicted uNeo levels to increase (1) during infections, (2) with increasing age, (3) over the gestation period and in estrous females; and (4) to vary seasonally. Our results showed uNeo levels varied over a one-year period and increased in individuals showing respiratory symptoms. Contrary to chimpanzees, uNeo levels did not vary with age or female reproductive status, possibly due to our small sample size. Our study provides a baseline for a better understanding of bonobo’s immunocompetence in the context of socio-ecological pressures and for monitoring the health of wild populations.

**Supplementary Information:**

The online version contains supplementary material available at 10.1007/s10393-023-01633-y.

## Introduction

Environmental pathogen exposure shapes and challenges the immune system of animals (Nunn et al., 2006; Brinkworth & Pechenkina, 2013). Species coevolve with their pathogens and minimize the cost of immune defense strategies by developing and maintaining immunocompetence (Zuk & Stoehr, 2002; Gandon et al., 2008; Brock et al., 2014), likely altered by environmental changes that shift and modify the incidence and prevalence of infectious diseases (Acevedo-Whitehouse & Duffus, 2009; Altizer et al., 2013; Paniw et al., 2022). This plasticity in immune functioning is a prerequisite to successfully adapt to changes in disease risk caused by changing environments in our increasingly globalized world (Capri et al., 2014). A better understanding of wildlife health is of major importance in the context of ecosystem change with ecoimmunology aiming to comprehend the plasticity of a host’s immunocompetence in the context of socio-ecological pressures (Adelman et al., 2014; Schoenle et al., 2015; Bowden et al., 2017).

Disease outbreaks can pose serious risks to the conservation and survival of endangered species (Ryan & Walsh, 2011). All great ape species are listed as endangered or critically endangered on the IUCN Red List (IUCN, 2021). Of concern is the transmission of infectious diseases, particularly from humans (Dunay et al., 2018), threatening wild populations, as documented for gorillas (*Gorilla* spp.) and chimpanzees (*Pan troglodytes*) (Leendertz et al., 2006; Köndgen et al., 2008; Grützmacher et al., 2016). In bonobos (*Pan paniscus*), little is known about infectious diseases so far (Behringer et al., 2018; Maibach & Vigilant, 2019; Yoshida et al., 2021). Only a few, mainly anecdotal reports are available from the wild, describing cases of flu-like and respiratory diseases apparently without detrimental impact (Sakamaki et al., 2009; Ryu et al., 2020). The paucity of published reports on infectious diseases in bonobos in comparison with chimpanzees is likely due to the scarcity of long-term study sites of the former. Moreover, symptoms of disease can be subtle and therefore hard to notice in wild individuals, particularly in social species (Lopes, 2014). This calls for a better understanding of non-invasive biomarkers to track immune responses in wild animals.

Non-invasive biomarkers of immune functioning can be used to investigate immunocompetence in wild animals (Murr et al., 2002; Negrey et al., 2021). Neopterin is a biomarker of the cell-mediated immune response, and its level typically increases during infections caused by viruses and intracellular bacteria and parasites (Hamerlinck, 1999; Berdowska & Zwirska-Korczala, 2001; Widner et al., 2002). Neopterin can be measured from non-invasively collected samples such as urine. Urinary neopterin (uNeo) has been used in several studies on non-human primates (González et al., 2020; Behringer et al., 2021; Negrey et al., 2021).

Neopterin levels in primates and humans typically vary with age, sex, and reproductive state and follow climatic and/or disease seasonality. Higher uNeo levels were reported in early- and late-life stages, in line with a shift from a mostly cell-mediated to a predominantly humoral immune response in immune ontogeny in bonobos (Behringer et al., 2021) and with inflammaging and immunosenescence in aging humans and Barbary macaques (Murr et al., 2003; Müller et al., 2017), respectively. Similarly, neopterin levels of humans and chimpanzees were higher in potentially fertile and pregnant females compared to lactating and cycling ones corresponding to hormonal fluctuation modulating immune function and to an increase in disease susceptibility in estrous and pregnant females (Boyunağa et al., 2005; Negrey et al., 2021). Increased uNeo levels were also found in response to low ambient temperatures (humans: Mohyuddin et al., 2017; chimpanzees: Löhrich et al., 2018), and increased malaria transmission rates in humans (Picot et al., 1993; also see Altizer et al., 2006). In humans, seasonal fluctuations of neopterin levels have been related to a variety of factors, including variations of the microbial environment (Picot et al., 1993), compromised immune functions (Nelson, 2004), and even the presence of allergens that induce a shift to a humoral immune response (Ledochowski et al., 2001; Pinto et al., 2007). Sex differences have been reported to influence uNeo levels in chimpanzees and bonobos, with males showing higher neopterin levels than females, which the authors associated with the effect of the immunomodulatory steroid hormone testosterone (Behringer et al., 2017; Negrey et al., 2021), although this sex difference was not found in other studies (Behringer et al., 2019; González et al., 2020). Urinary neopterin measurements in wild infant and juvenile bonobos showed sex-specific patterns of cell-mediated immune ontogeny, with males having higher uNeo levels until 3 yo and females having higher levels from 6 to 8 yo. These patterns did not differ from zoo-housed individuals suggesting an independence from environmental conditions (Behringer et al., 2021).

In summary, studies on bonobos showed higher uNeo levels in juvenile compared to adult wild individuals (Behringer et al. 2021) and higher uNeo levels in adult males compared to adult females in zoo individuals (Behringer et al. 2017). So far, it is unknown which factors influence the variation in uNeo levels in adult wild bonobos and how this compares to what we know from closely related species like chimpanzees and humans. We used uNeo levels as a proxy for cell-mediated immunity of wild bonobos investigating its variability and the effect of potential pathogens, age, sex, season, and reproductive state. Based on the findings in humans, chimpanzees, and bonobos, we predicted uNeo levels in wild bonobos to **1/** increase during infections; **2/** vary seasonally; **3/** increase with age; and **4/** increase over the gestation period and in potentially fertile females.

## Materials and Methods

### Ethics

All methods applied were strictly non-invasive and noncontact as required by the IUCN guidelines. The Institut Congolais pour la Conservation de la Nature (ICCN) granted the permission to conduct research at LuiKotale, Salonga National Park, Democratic Republic of the Congo (DRC). The research project was approved by the ethics committee of Liverpool John Moores University (LJMU).

### Study Site and Study Subjects

Data were collected between 2010–2011 and 2016–2019 at the LuiKotale field site (2°45′36’’S; 20°22′43’’E; Hohmann & Fruth, 2003), located west of the southern block’s border of Salonga National Park, DRC. Two bonobo communities were habituated to the presence of human observers, namely the Bompusa West (BpW) and the Bompusa East (BpE) community. In January 2020, BpW and BpE communities were composed of 23 (16 females, 7 males) and 16 (10 females, 6 males) sexually mature individuals, respectively.

During focal follows, individuals were visually assessed for the presence of sickness behaviors (i.e., asthenia, anorexia) and symptoms of infectious and/or other diseases for at least 15 min. We recorded activity level, appetite (approximate feeding rate and time spent feeding compared to neighbors), locomotion, presence/absence of diarrhea, repetitive respiratory symptoms (e.g., cough, sneeze), and injuries. Bonobo age was based on the project’s long-term records and reflects estimates based on an individual’s life history (e.g., number and age of offspring in the community; social rank) and morphological features. Age estimates at the time of sample collection ranged from 10 to 42 years (Table S1; S2). Females’ sexual swellings, reflecting the likelihood of their ovulation’s timing (Douglas et al., 2016), were assessed during follows and scored into four categories from 1 (not swollen) to 4 (fully swollen) following Furuichi (1987).

### Climatological Data

We retrieved climatological data for 2010–2011 and 2016–2019 from the LuiKotale long-term records. Daily cumulative rainfall was measured using a rain gauge (mm/m^2^) open to the sky. Temperatures were measured using a min–max thermometer and a Bresser 5 in 1 Weather station deployed in the forest. We modeled climatic conditions at LuiKotale using Walter–Lieth climatic diagrams (Walter & Lieth, 1967), characterizing monthly moisture conditions based on the relationship between cumulative monthly rainfall and mean monthly temperatures. Months are classified as “wet” when monthly rainfall exceeded 100 mm; “transient” when monthly rainfall was ≤ 100 mm; and “dry” when the rainfall figures below the mean temperature line.

### Urine Sample Collection

Between December 2018 and October 2019, individual urine samples were collected every 1–2 months whenever possible, either directly into a folded leaf or indirectly from the surrounding vegetation or the ground (Surbeck et al., 2012). Urine was pipetted into 2-ml cryogenic tubes, marked, and protected from sunlight and body heat to avoid neopterin degradation (Behringer et al., 2017). In camp, cryotubes were transferred into liquid nitrogen and shipped frozen to the Max Planck Institute for Evolutionary Anthropology in Leipzig, Germany, where they were stored at  − 20°C. We added additional samples collected opportunistically between December 2012 and October 2018 and some in connection to a respiratory outbreak of the BpW community between December 2010 and September 2011 using the same methodology as described above. All cycling females were tested for their reproductive state monthly using urinary pregnancy tests (Artron Hcg-Test sticks). The pregnancy stage was assessed retrospectively based on the offspring’s birthdate.

### Urinary Neopterin Measurement

Frozen urine samples were shipped on dry ice to the German Primate Center in Göttingen, Germany. All samples, controls, and standards were thawed to room temperature, vortexed, and measured in duplicate with a commercial competitive neopterin ELISA kit (Neopterin ELISA, Ref. 59,321, IBL International GmbH, Germany) validated for bonobo urine samples (Behringer et al., 2017). Inter-assay variation was 6.74% (*n* = 10) and intra-assay variation was 3.66% (*n* = 309). To account for variation in hydration status, the urine’s density (urine specific gravity, SG) was measured using a digital handheld refractometer (TEC +  + Dr. Volker Schmidt GmbH: TR35U). All samples with a SG < 1.003 (*n* = 2) and > 1.05 (*n* = 3) were removed from the dataset to avoid over- or underestimating the uNeo level (Emery Thompson et al., 2016). To assure that most samples were within the range of the neopterin assay’s standard curve, SG was used to determine dilution factors between 1:10 and 1:400 for each sample. Samples that fell outside of the measurement range were remeasured at an appropriate dilution factor. Final urinary neopterin levels are expressed in nmol/L corrected for SG and were calculated following Miller et al. (2004), by correcting for dilution factor, and individual and population urine density (SG_pop_ = 1.015).

### Statistical Analyses

All models were fitted in R 4.0.3 (R Core Team, 2020). We ran a linear mixed-effect model (LMM) using the lme function from the “nlme” package in R (Laird & Ware, 1982) to assess the effects of age, sex, health status, and season, on the uNeo levels (m1**;** *n* = 293). We decided against modeling rainfall and temperature to account for seasonality because of (1) collinearity and autocorrelation issues and (2) climatic variation not being as pronounced at LuiKotale as at other study sites where individual’s uNeo level varied following climatic changes (Löhrich et al., 2018; Kreyer et al., 2021). To account for potential seasonal variation in uNeo levels, we added the sine and cosine of the collection date converted into a continuous circular variable. The sine and cosine predictors represent a generic seasonal term and allow to model a wave-like periodic pattern throughout a year (Stolwijk et al., 1999). Finally, we added community and sample collection time as controls for inter-community differences and circadian rhythm of the uNeo level (Auzéby et al., 1988), respectively. We included the day and year of the sample collection per individual as nested random effects to account for multiple samples collected for the same individual on the same day but unevenly between years in the study period. We checked for the absence of collinearity between predictors using the check_collinearity function from the “performance” package (Lüdecke et al., 2021; VIF ≤ 1.24 for all predictors). We found a slight kurtosis and heteroskedasticity from the qq-plot of the residuals of the expected values versus observed values that can be explained by the failure of our model to predict the few very low and very high values (Figure S2).

To prevent redundancy between sex and reproductive state predictors, we ran a separate LMM (m2) with female only samples (*n* = 220) including reproductive state with the following levels: lactating, pregnant, post-reproductive, and two states of cycling with swelling stages (i) 1–2 and (ii) 3–4, as a predictor and keeping all other factors from (m1) as controls. In female bonobos, ovulation mostly occurs when swellings are at stage 3 or 4 (Douglas et al., 2016). In (m2), we also found no correlation between predictors (VIF ≤ 2.10 for all predictors). Table S3 summarizes all variables used in (m1) and (m2).

The significance of the full model to the null model comparison was derived using a likelihood ratio test (ANOVA with argument test set to “Chisq”; Dobson & Barnett, 2008). Null models only contained control variables and random effects in the respective models. In both models, the response variable uNeo was log-transformed and all other quantitative variables were z-transformed to a mean of 0 and a standard deviation of 1 (Schielzeth, 2010). Assumptions of normally and homogenously distributed residuals were met in both models, and diagnostic plots to verify whether there was no concerning impediment were run.

## Results

**Figure 1:**
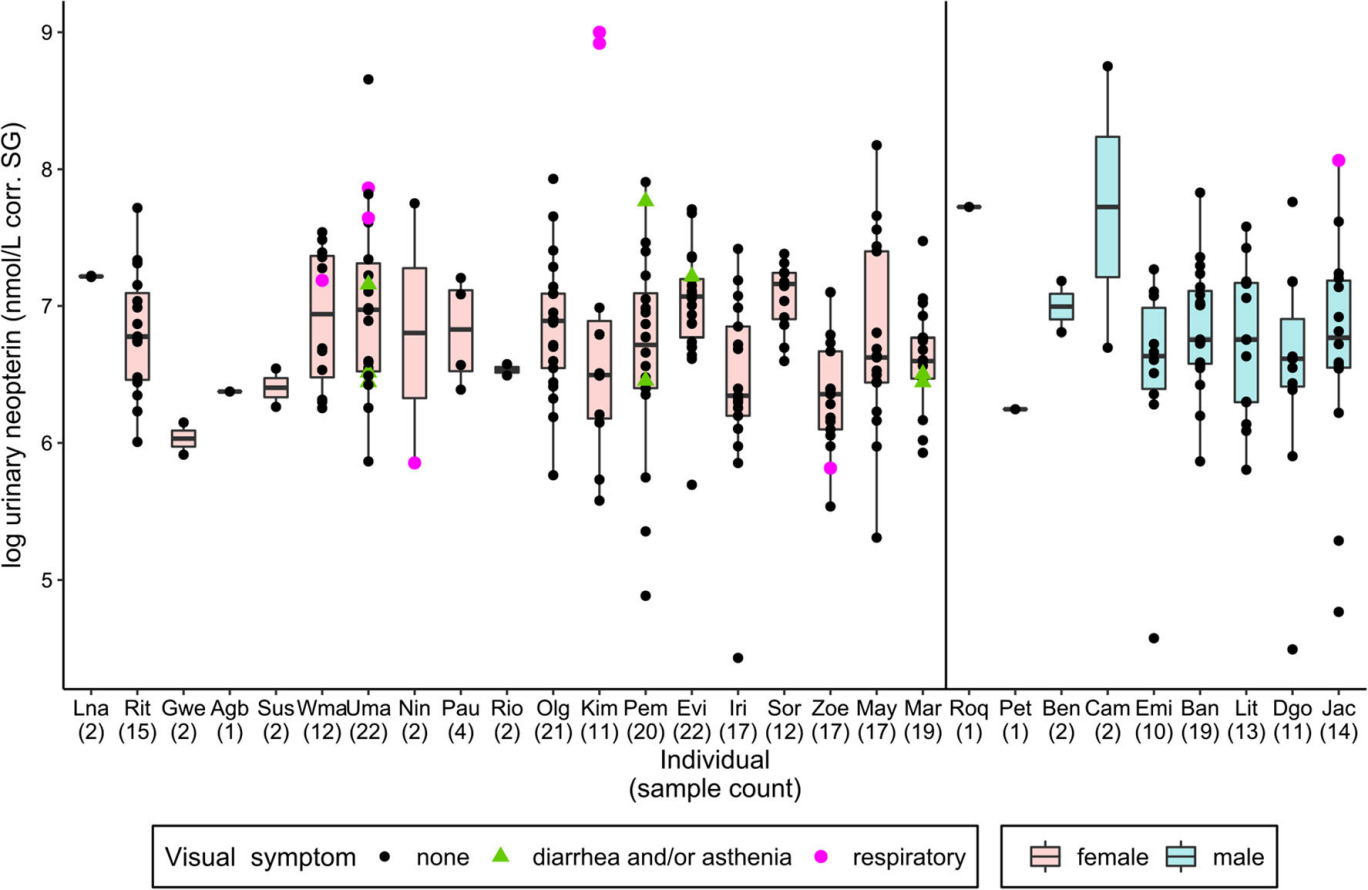
Log-transformed urinary neopterin (uNeo) level of LuiKotale bonobos between 2010 and 2019. Samples (*n* = 293) of individuals showing respiratory symptoms (pink circles), diarrhea and/or asthenia (green triangles) and asymptomatic individuals (black circles) are ordered by sex (red boxplot: female and blue boxplot: male), and within sex by mean individual estimated age on the sampling date. Numbers in brackets show sample count per individual. Boxplots’ show median (horizontal line), with 3rd and 1st quartiles (upper and lower limit of the box) and range (vertical line) (Color figure online).

Overall, 309 urine samples (230 female and 79 male samples) from 28 individuals (mean = 11 samples/individual, SD = 8.1; Table S1) collected in December 2010–September 2011 (*n* = 37), December 2016–October 2018 (*n* = 55), and December 2018–October 2019 (*n* = 217) were analyzed. Of these, samples from injured individuals (*n* = 16) were excluded from analysis, as injuries can also cause an increase in the uNeo level (González et al., 2020). We chose to remove samples from visually injured individuals as we considered the injury’s severity difficult to assess. In addition, the relation between injury/wound severity and neopterin level increase is unknown. Among 309 samples, 27 were collected from pregnant females (*n* = 5). We collected eight samples from six individuals showing signs of respiratory infection and eight samples from four individuals with symptoms of diarrhea and/or asthenia. Levels of uNeo varied between 83.9 and 8,100.9 nmol/L corr. SG (median = 832.4; *n* = 293 samples). In uNeo levels, we found inter-individual variation between individuals (ANOVA: *F* value = 1.55, *p*-value < 0.05, *n* = 293; Fig. 1).

**Figure 2:**
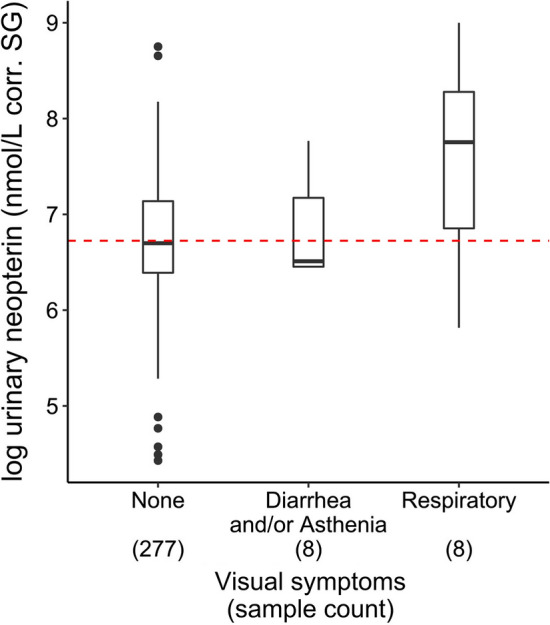
Log-transformed urinary neopterin (uNeo) level of LuiKotale bonobos between 2010 and 2019 in the presence or absence of visual symptoms. Numbers in brackets are the sample count per category. Red dashed line: median of uNeo levels across all samples (*n* = 293). Boxplots show median (horizontal line), with 3rd and 1st quartiles (upper and lower limit of the box), range (vertical line) and outliers (black dots) (Color figure online).

In m1, the full-null model comparison was significant (χ^2^ = 30.5, df = 5, *p*-value < 0.001). Only the presence of symptoms (Fig. 2) and the sine function were significant predictors of uNeo levels (Table 1). Bonobos with respiratory symptoms had higher uNeo levels compared to visually asymptomatic individuals (ANOVA: *F* value = 6.65, *p*-value < 0.01; post hoc test Tukey HSD for respiratory symptoms–no symptom: *p*-value < 0.001). However, some individuals showed elevated uNeo levels (*n* = 12) despite being visually asymptomatic (max _asymptomatic_ = 6,316.2 nmol/L corr. SG; *n* = 278).

**Table 1:** Model output for fixed effects in (m1) testing for the effect of age, sex, visual symptoms, and season on the urinary neopterin level (log-transformed).

	Estimate	Std. error	DF	*t*-value	*p*-value
(Intercept)	6.760	0.073	178	92.566	0.0000
Age (z-transformed)	− 0.055	0.049	49	− 1.121	0.2677
Sex	− 0.070	0.108	25	− 0.643	0.5258
Visual symptoms	0.590	0.189	49	3.118	**0.0030**
Sine (date)	0.218	0.052	49	4.153	**0.0001**
Cosine (date)	− 0.045	0.054	49	− 0.835	0.4077

Bold values indicate significant

**Figure 3:**
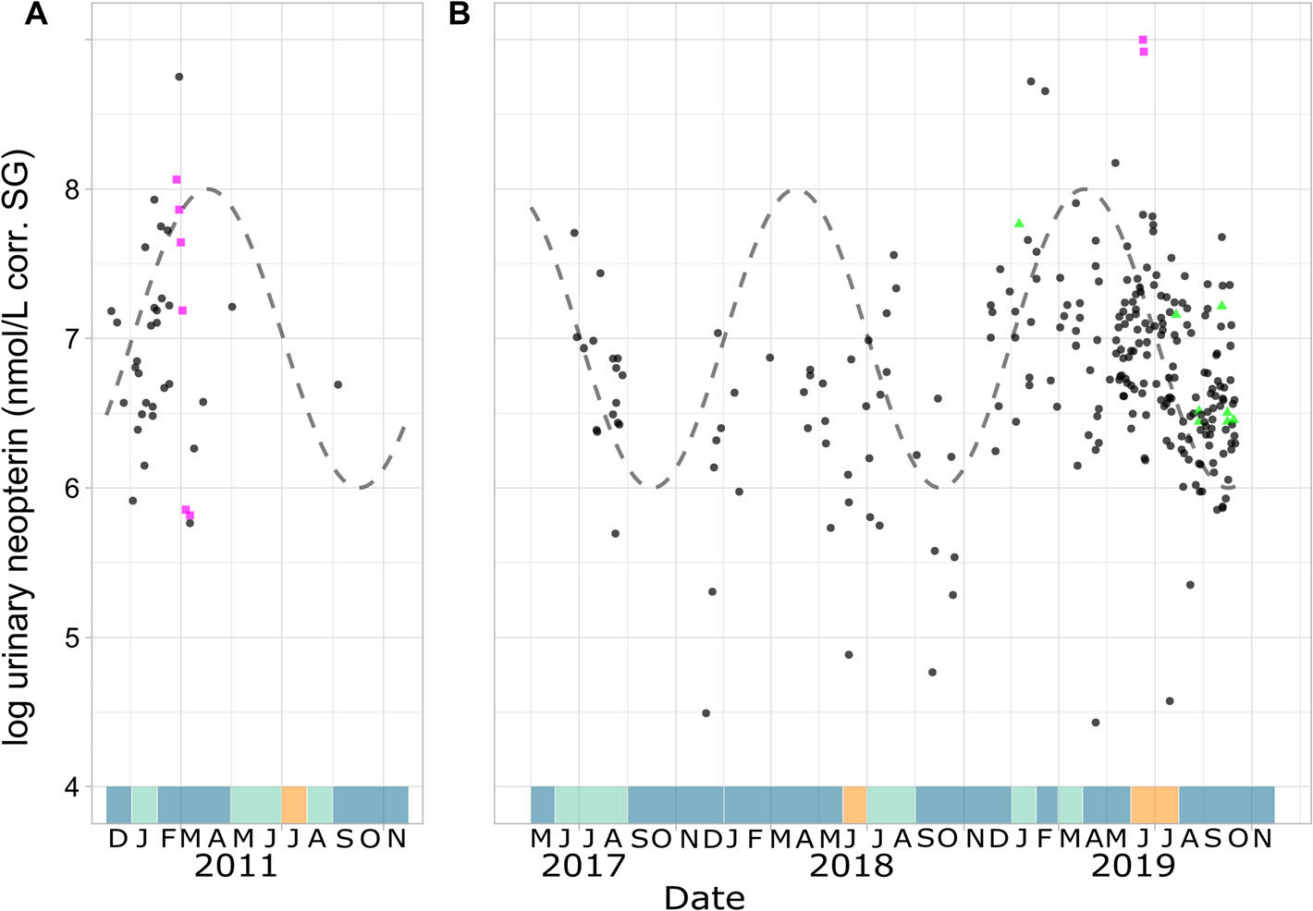
Log-transformed urinary neopterin (uNeo) level in samples collected from **A** 2011 and **B** 2017 to 2019 (*n* = 292). Dots represent uNeo measurements from visually asymptomatic (black circles), symptomatic with unknown infection (green triangles) and symptomatic with respiratory infection (pink rectangles) individuals. Dashed line represents one year period sinusoidal oscillation. Areas above the x axis colored by month represent dry (orange), transient (turquoise) and wet (blue) months as defined by the Walter–Lieth climate diagrams (Figure S1) (Color figure online).

According to our model (m1), uNeo levels fluctuated seasonally following a one-year oscillation period, with higher uNeo levels in March–April and lower levels in September–October. This pattern seems to be unrelated to the climatic seasonality observed at the study site (Fig. 3). The Walter–Lieth climatic diagrams showed seasonality in LuiKotale was not pronounced (Figure S2) with 6.45% of the study months (*n* = 31) being dry, 32.3% being transient, and 61.3% being wet, suggesting the absence of a prolonged dry season between the two annual rainy seasons.

For the second model (m2), the full-null model comparison was not significant (χ^2^ = 7.1, df = 4, *p*-value = 0.13), indicating that variation in female uNeo levels was not explained by the reproductive state in our samples (Figure S3).

## Discussion

In this study, we analyzed uNeo levels as a proxy for the activity of cell-mediated immunity in relation to seasonality and individual’s age, sex, reproductive status, and presence of visual symptoms indicative of respiratory infections in wild bonobos. We found that urinary neopterin levels were elevated in individuals with symptoms of respiratory infection, and showed a yearly seasonal pattern, supporting our first and second predictions. However, prediction (3) was not supported, as, in contrast to chimpanzees (Negrey et al., 2021), uNeo levels in bonobos were not related to age. Our focal individuals were adults only, and estimated ages may have caused a range of error potentially concealing a tendency. The oldest female was estimated to be around 42 y, the oldest male around 32 y at the time of sampling. Although life expectancy in wild bonobos is not yet well documented, it is thought to be around 45–50 y (Furuichi, 2019); therefore, our sample size of older individuals could have been too small to detect an age effect on the uNeo level as pointed out in Negrey et al. (2021). Similarly, prediction (4) was also not supported, with female’s reproductive state not being related to uNeo levels, in contrast to other studies on humans and chimpanzees (Bichler et al., 1983; Negrey et al., 2021). Nevertheless, the trends we observed pointed toward the expected direction, with pregnant and fertile females showing higher uNeo levels compared with females at other reproductive stages. We can add that female bonobos were not sampled across all reproductive states, making intra- and inter-individual comparisons limited, and possibly explaining, along with small sample sizes, the absence of the usually robust increase in uNeo level during pregnancy.

In line with findings from humans and chimpanzees, uNeo levels were higher in bonobos with respiratory symptoms, compared to asymptomatic individuals (Denz et al., 1990; Wu et al., 2018a). However, we also found elevated uNeo levels in samples (*n* = 12) from visually asymptomatic individuals, possibly caused by an underlying infection (Eisenhut, 2013). Infections can cause an increase in neopterin production in humans preceding the onset of symptoms by 24–48 h (Giovannoni et al., 1997; Murr et al., 2002). In order to verify this observation in wild bonobos, we would need to follow individuals for up to two days after collection of the urine sample, allowing to record the appearance of potential symptoms. Individuals with non-respiratory symptoms such as asthenia and diarrhea did not differ in their uNeo levels from visually asymptomatic individuals. The immune reaction that increases neopterin levels after infection is specific to intracellular pathogens such as viruses and malaria parasites (Fuchs et al., 1988). Gastrointestinal symptoms can also be caused by extracellular pathogens that do not activate this pathway of cell-mediated immunity and thus do not result in elevated neopterin levels (Plata-Nazar et al., 2019). Gastrointestinal parasites, including *Oesophagostomum* spp. and *Trichuris* spp., have been identified in fecal samples from LuiKotale bonobos (Kreyer et al., 2021) and are often associated with diarrhea and weakness (Huffman et al., 1996; Krief et al., 2008). Therefore, we suggest gastrointestinal parasitic infections as a possible explanation to our findings, supporting the specificity of uNeo as a biomarker for intracellular infections.

Our results showed uNeo levels to follow a seasonal pattern with a one-year periodicity. Urinary neopterin levels were highest at the beginning of the short rainy season around March–April and lowest at the start of the long rainy season in September–October. This pattern is different from the seasonal pattern observed in uNeo levels of chimpanzees in West Africa. In Taï chimpanzees, Côte d’Ivoire, Löhrich et al. (2018) found the highest uNeo levels during the long dry season, when the minimum ambient temperatures were decreasing, and the lowest uNeo levels at the end of the rainy season (Anderson et al., 2006). One explanation for uNeo seasonality could be underlying seasonality in the prevalence of viral respiratory diseases. In the tropics, respiratory infections, such as the respiratory syncytial virus or influenza, are more prevalent during rainy seasons (Shek & Lee, 2003; Tamerius et al., 2013). However, the few cases of symptomatic respiratory disease we observed in wild bonobos occurred during a short rainy (February–March 2011) as well as during a dry season (June 2019; Figure S4), suggesting that, at our field site, the seasonality of uNeo levels detected by our model is not linked to seasonally fluctuating climatic conditions. Moreover, in the 20 years of observation at LuiKotale, only one respiratory outbreak of non-anthropogenic origin (Leendertz *pers. comm*.) was observed (BF unpublished data), suggesting that respiratory infections are not very prevalent in these communities and that they are therefore an unlikely candidate to explain the observed seasonal variation in uNeo levels.

Seasonal fluctuations in tropical rainfall can increase the abundance of vectors with aquatic larval stages such as mosquitoes that transmit vector borne diseases including malaria (Altizer et al., 2006). Wu et al. (2018b) showed that malaria infections occurred seasonally in one community of Taï chimpanzees, where malaria detection rates were highest three months after the start of the rainy season and lowest at the end of the dry/beginning of the rainy season. This pattern resembles the seasonality of uNeo levels observed in our study area, where malaria affects the human population (Muganza et al., 2012; Tajudeen & Van Heerden, 2019). Rate and timing of malaria infections are not well documented in wild bonobos; however, malaria seems to be less prevalent than in wild chimpanzees (Liu et al., 2017; Loy et al., 2017). In one bonobo field site, the Tshuapa–Lomami–Lualaba area, a high prevalence of infections caused by *Laverania* (a subgenus of the genus *Plasmodium*) was found in bonobos, but not at 10 other field sites (Liu et al., 2017). Furthermore, an increase in the neopterin level was associated with the severity of malaria infection in humans (Brown et al., 1990; 1991; te Witt et al., 2010). We suggest bonobos do not develop severe forms of malaria at our study site, perhaps because of (1) the *Plasmodium*/*Laverania* species transmitted by mosquitoes in the area being less virulent compared to other species or (2) the fact bonobos possess an allotype of their major compatibility complex (MHC) class-I that plays a role in antigen recognition, resembling that providing a resistance against malaria in humans and is positively associated with milder forms of malaria infections (Hill et al., 1991; Nikolich-Žugich et al., 2004; de Groot et al., 2018). If indeed MHC class-I allotypes in bonobos provide protection against severe forms of malaria infection, both the seasonal fluctuation in uNeo levels and the absence of a drastic increase in uNeo levels can be explained. To test this hypothesis, future studies should (1) test for the prevalence of malaria infection and (2) identify the MHC class-I allotypes carried by individuals in the studied population.

Seasonality of the uNeo level variation could also be explained by other infectious diseases potentially present in the study area, such as monkeypox (Mandja et al., 2019) or measles (Martinez, 2018). They are known to occur during the dry months, with an irregular or absent annual cyclicity (Martinez, 2018; Mandja et al., 2019). Further studies are necessary to identify pathogens that affect bonobos at LuiKotale and the uNeo response they elicit. Also, testing seasonality over longer periods of time than one year may uncover trends possibly matching the cyclicity of infectious diseases other than those mentioned above. The seasonal fluctuation of the uNeo levels reported in our study could thus reflect specific conditions of our study population, such as the prevalence, frequency, and type of pathogens.

Antiviral antibodies for human respiratory viruses, including parainfluenza viruses, respiratory syncytial virus, rhinovirus, and mumps virus, were detected in several bonobo populations (Yoshida et al., 2021), highlighting the threat from anthropozoonoses. LuiKotale bonobos are isolated from human settlements with researchers being the only humans in regular proximity to the communities under study. Researchers wear surgical masks when following bonobos to limit transmission of pathogens. Therefore, it is very unlikely that LuiKotale bonobos encounter human borne novel pathogens to which their immune system is completely naive, while captive conspecifics are regularly exposed and show a high sensitivity to human transmitted pathogens (see review in Stevens, 2020). This is in line with studies on wild and zoo-housed chimpanzees (Negrey et al., 2019; Glasser et al., 2021). Examining pathogen susceptibility and prevalence in wild bonobos will require integrating neopterin measurements with local disease ecology. Nevertheless, human pathogen transmission remains a serious threat for Africa’s wild great ape populations (Leendertz et al., 2006; Boesch, 2008; Fruth et al., 2016), and understanding baseline levels and factors triggering deviation from those baseline levels is crucial for future population management.

In conclusion, our study supports the use of uNeo levels as a marker of cell-mediated immune response, being an efficient way to monitor outbreaks of infectious diseases most likely caused by viruses and other intracellular pathogens. Our results showed that uNeo levels were more elevated in individuals with symptoms associated with respiratory infections and cyclically at the beginning of the short rainy season, but uNeo levels were not elevated in older individuals or pregnant and fertile females. Our results revealed a yearly seasonality in the variation of the uNeo levels potentially related to the seasonality in the prevalence of malaria and/or other infectious diseases, but unrelated to climatic fluctuations that were found to underlie seasonal uNeo patterns in other species, including chimpanzees. Disease monitoring in wild populations of bonobos and other great apes is crucial, as rapid environmental changes are increasingly affecting the pathogenic environment of wild populations. We recommend systematizing the use of uNeo levels as health monitoring tool of individual habituated great apes, considering the influence of the different individual and environmental factors discussed. Although our study is limited to one population only, our results shed light on some important aspects of bonobo cell-mediated immunity and encourage the incorporation of more bonobo populations and inter-specific comparative studies.

## Supplementary Information

Below is the link to the electronic supplementary material.

Supplementary file1 (DOCX 930 KB)
