# Supplementary material for: Neopterin Levels in Bonobos Vary Seasonally and Reflect Symptomatic Respiratory Infections
Source: Ecohealth. 2023 May 15;20(1):93–104. doi: 10.1007/s10393-023-01633-y (PMC10257626; doi:10.1007/s10393-023-01633-y)
Supplement: Supplementary file 1 — Supplementary file1 (DOCX 930 KB) [file 10393_2023_1633_MOESM1_ESM.docx]

**Supplementary information:**

Table S1: Sex (F: Female, M: Male), sample count and mean age estimate and standard error for individuals (3 letters code) sampled in (m1)

| **Individual (3 letters code)** | **Sex** | **Sample count** | **Mean age estimate [years (sd)]** |
| --- | --- | --- | --- |
| Lna  Rit  Gwe  Agb  Sus  Wma  Uma  Nin  Pau  Rio  Olg  Kim  Pem  Evi  Iri  Sor  Zoe  May  Mar  Roq  Pet  Ben  Cam  Emi  Ban  Lit  Dgo  Jac | F  F  F  F  F  F  F  F  F  F  F  F  F  F  F  F  F  F  F  M  M  M  M  M  M  M  M  M | 2  15  2  1  2  12  22  2  4  2  21  11  20  22  17  12  17  17  19  1  1  2  2  10  19  13  11  14 | 12.0 (0)  13.7 (0.6)  17.0 (0)  19.0  19.0 (2.8)  19.7 (3.1)  23.5 (3.2)  26.0 (0)  27.0 (0)  27.0 (0)  28.2 (2.4)  28.4 (0.7)  29.5 (0.7)  33.5 (0.7)  34.0 (2.6)  35.8 (0.4)  35.9 (2.6)  39.4 (0.7)  41.2 (2.5)  10.0  11.0  16.5 (0.7)  19 (0)  19.3 (3.6)  21.5 (0.8)  21.5 (0.7)  24.3 (0.9)  30.6 (2.8) |

Table S2: Sample count by age class (in years) and sex based on age estimates of the individual at the time of sample collection used in (m1).

| Age class (years) | [10-15[ | [15-20[ | [20-25[ | [25-30[ | [30-35[ | [35-40[ | [40-42[ | **Total** |
| --- | --- | --- | --- | --- | --- | --- | --- | --- |
| Female | 19 | 8 | 13 | 69 | 36 | 49 | 26 | **220** |
| Male | 4 | 4 | 47 | 6 | 12 | 0 | 0 | **73** |
| **Total** | **23** | **12** | **60** | **75** | **48** | **49** | **26** | **293** |

Table S3: Description of variables used in model 1 (m1) looking at the effect of individual age, sex, presence of symptoms and season on the log (urinary neopterin level) and model 2 (m2) tesing the effect of the reproductive status of female on the log (urinary neopterin level) in bonobos. Type of variable: N= numeric; F= Factor.

| Model | Variable class | Variable name | Type of variable | Transformation |
| --- | --- | --- | --- | --- |
| m1 (n= 293) | Response | uNeo | N | Log-transformed |
|  | Fixed | Age | N | Z-transformed |
|  |  | Sex | F |  |
|  |  | Visual symptoms | F |  |
|  |  | Sine (date) | N |  |
|  |  | Cosine (date) | N |  |
|  | Control | Collection time | N | Z-transformed |
|  |  | Community | F |  |
|  | Random | ID/Year/Day | F |  |
| m2 (n= 220) | Response | uNeo | N | Log-transformed |
|  | Fixed | Reproductive status | F |  |
|  | Control | Age | N | Z-transformed |
|  |  | Visual symptoms | F |  |
|  |  | Sine (date) | N |  |
|  |  | Cosine (date) | N |  |
|  |  | Collection time | N | Z-transformed |
|  |  | Community | F |  |
|  | Random | ID/Year/Day |  |  |


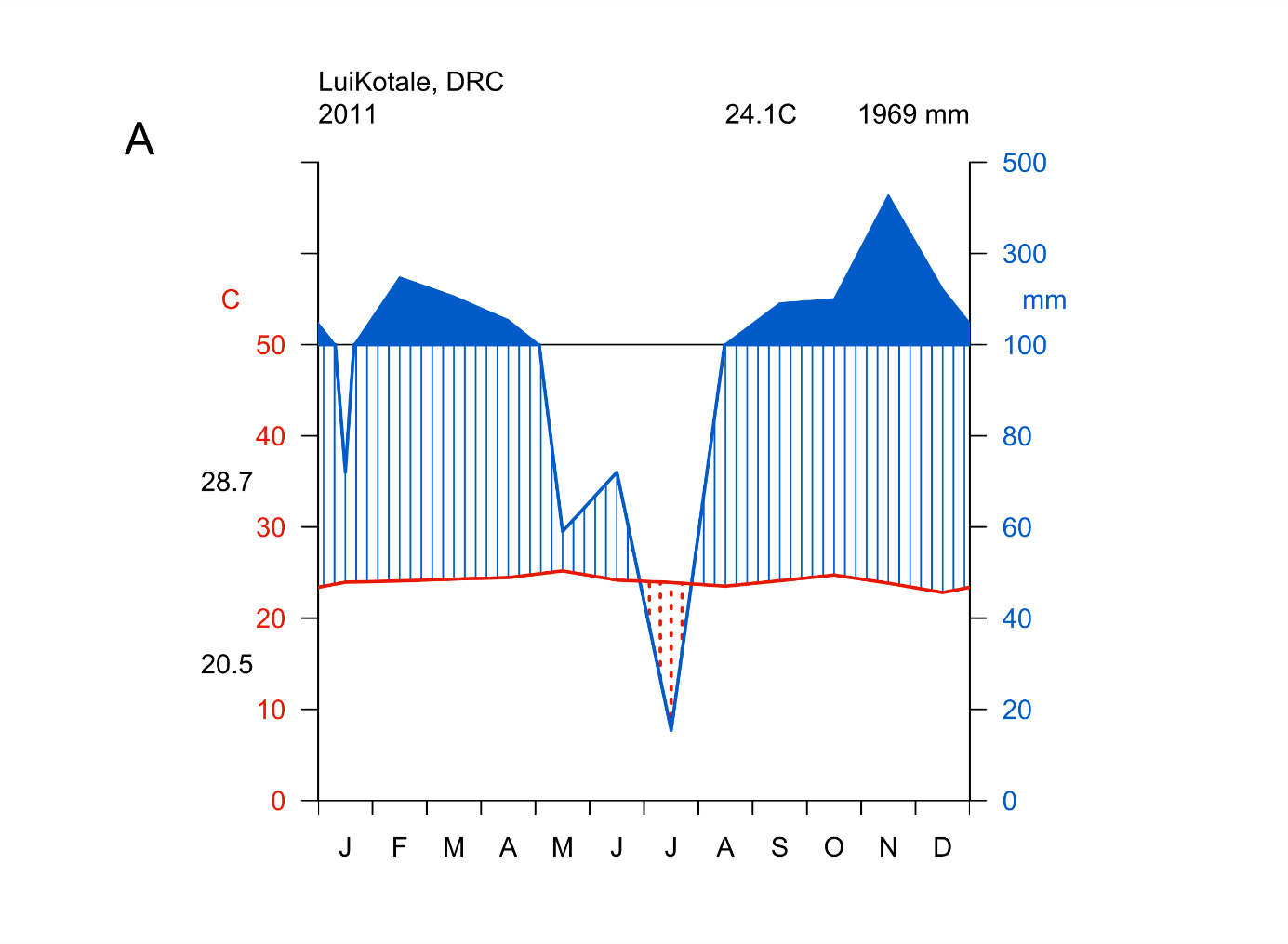

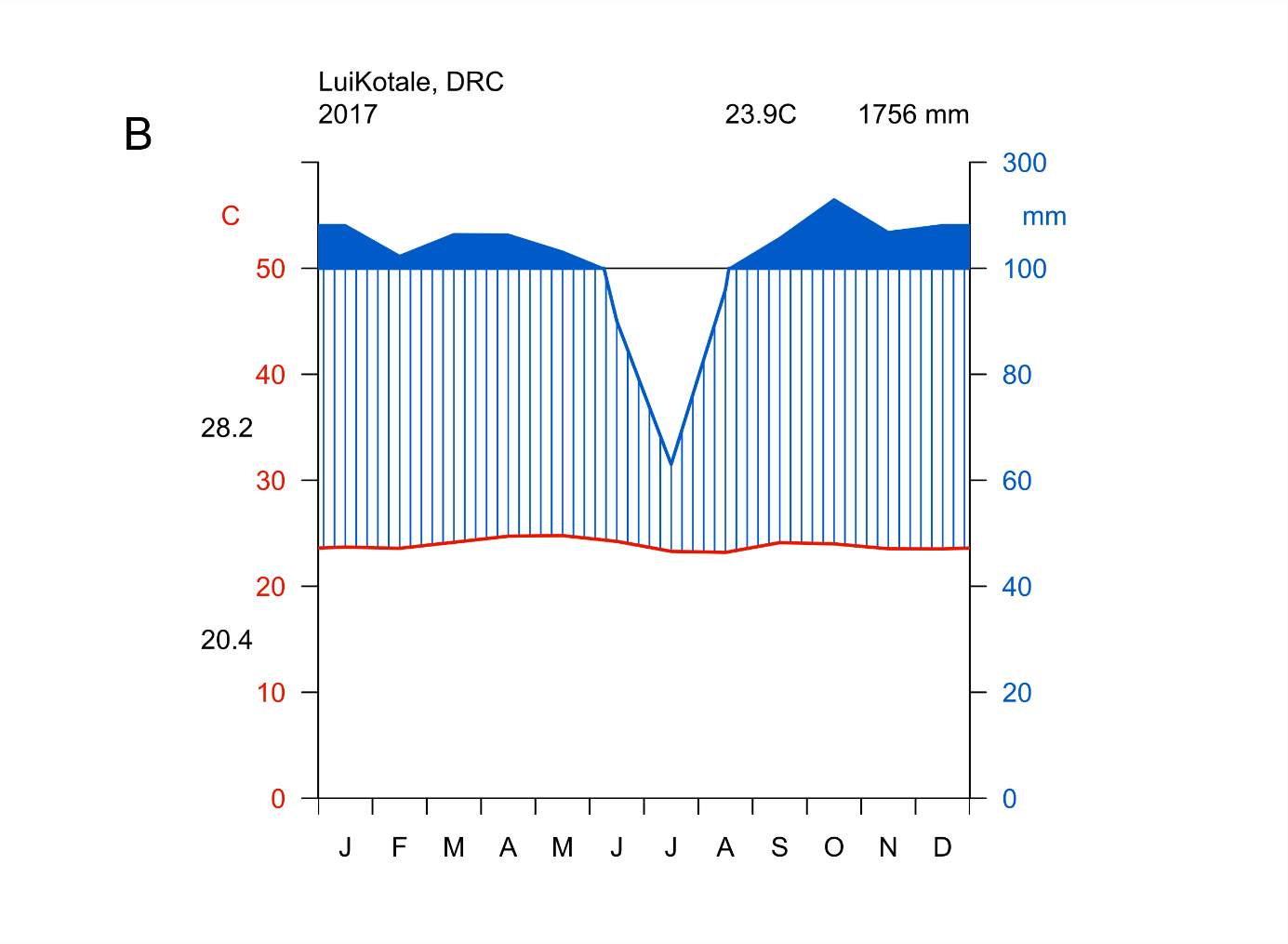


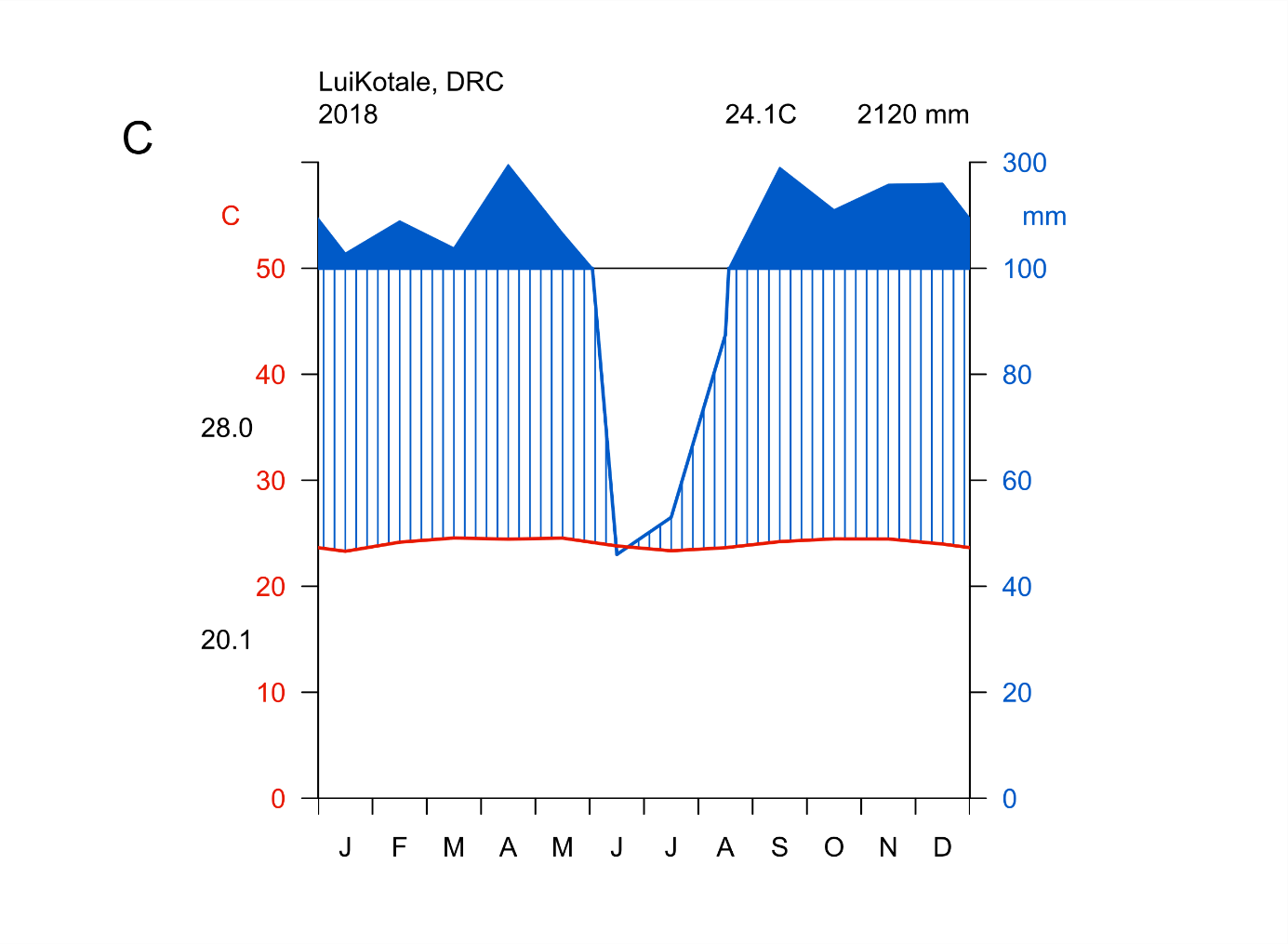

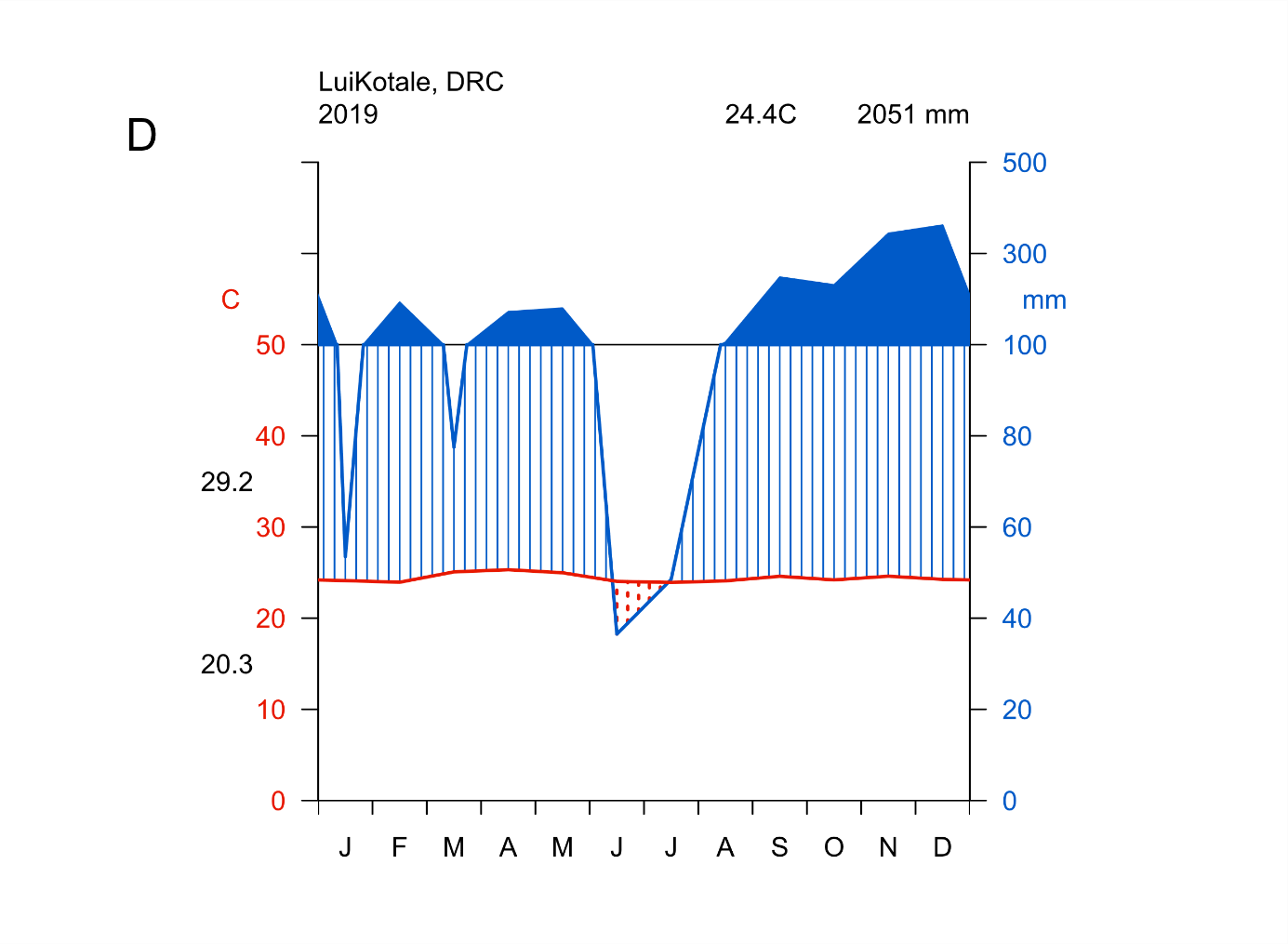


Figure S1: Walter-Lieth climate diagrams in A) 2011, B) 2017, C), 2018 and D) 2019 based on averaged monthly temperatures and rainfall data collected at LuiKotale, DRC. Red line: mean monthly temperature; blue line: monthly cumulative rainfall. Months are classified as “wet” when monthly rainfall is > 100 mm; “transient” when monthly rainfall is ≤ 100 mm; and “dry” when the rainfall figures below the mean temperature line (Walter and Lieth, 1960)


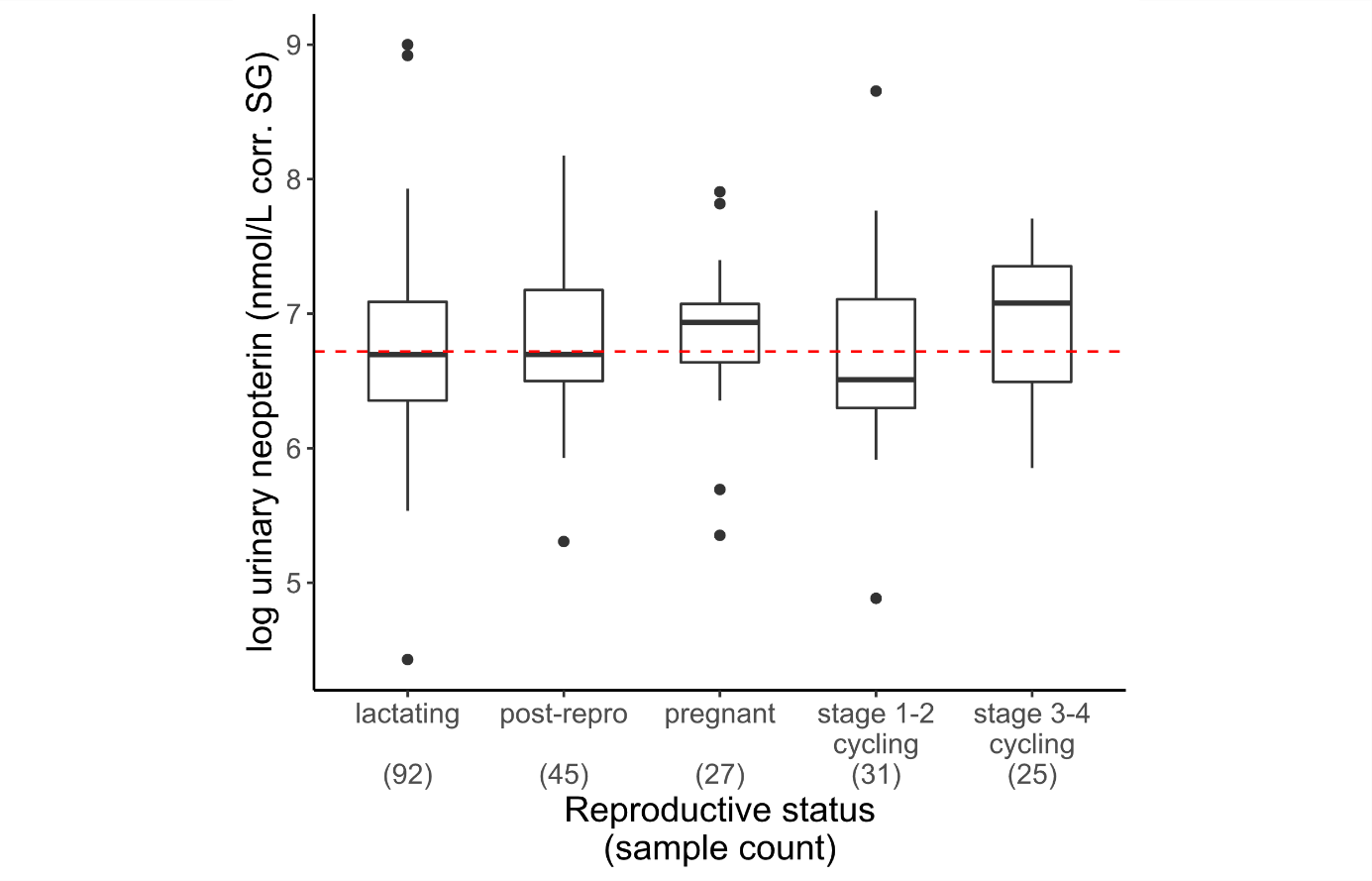


Figure S2: Log-transformed urinary neopterin level (uNeo) of LuiKotale bonobos between 2011 and 2019 in different reproductive states. Boxplots show median (horizontal line), with 3^rd^ and 1^st^ quartiles (upper and lower limit of the box), range (vertical line) and outliers (black dots). The dashed line represents the median of the log(uNeo) levels.
